# Endobariatric Management of Metabolic Dysfunction-Associated Steatotic Liver Disease: A Narrative Review

**DOI:** 10.3390/biomedicines14020345

**Published:** 2026-02-02

**Authors:** Muaaz Masood, Reem Z. Sharaiha, Asma Siddique, Shanley Deal, Richard A. Kozarek

**Affiliations:** 1 Department of Gastroenterology and Hepatology, Center for Digestive Health, Virginia Mason Franciscan Health, Seattle, WA 98101, USA; muaazm@gmail.com (M.M.);; 2 Department of Gastroenterology and Hepatology, Weill Cornell Medicine, New York Presbyterian, New York, NY 10065, USA; 3 Department of General and Bariatric Surgery, Center for Weight Management, Virginia Mason Franciscan Health, Seattle, WA 98101, USA; 4 Center for Interventional Immunology, Benaroya Research Institute, Virginia Mason Franciscan Health, Seattle, WA 98101, USA

**Keywords:** endobariatric and metabolic therapy, metabolic dysfunction-associated steatotic liver disease, MASLD, MASH

## Abstract

As the rates of type 2 diabetes and obesity have increased globally, the prevalence of metabolic dysfunction-associated steatotic liver disease (MASLD), previously termed non-alcoholic steatotic fatty liver disease (NAFLD), has risen concomitantly worldwide. MASLD is now the most common etiology of chronic liver disease and is the leading indication for liver transplantation in the United States. Patients with MASLD have an increased risk of progression to metabolic dysfunction-associated steatohepatitis (MASH), cirrhosis, hepatocellular carcinoma, extrahepatic malignancies, as well as liver- and cardiovascular-related mortality. Diet and lifestyle modifications with a goal of ≥10% total body weight loss—required to reverse steatosis, steatohepatitis, and fibrosis—are often challenging and ineffective. Although novel pharmacotherapies have recently been approved and others are in development, cost, adherence, and adverse effects remain potential limitations. Bariatric surgery, including Roux-en-Y gastric bypass and sleeve gastrectomy, is highly efficacious and a cost-effective treatment for obesity and associated medical problems. However, bariatric surgery may be associated with morbidity and mortality. Endoscopic bariatric and metabolic therapy (EBMT) has recently emerged as a promising treatment modality and offers an alternative to surgery. Primary EBMTs include intragastric balloon placement, aspiration therapy, endoscopic sleeve gastroplasty, duodenal mucosal resurfacing, duodenal–jejunal bypass liner, and primary obesity surgery endoluminal (POSE 2.0). Secondary EBMTs include transoral outlet reduction, argon plasma coagulation of the anastomosis, and revisional endoscopic sleeve procedure. We review the recent literature on primary EBMTs and secondary EBMTs for the treatment of obesity and MASLD, the pathophysiologic mechanisms, efficacy, safety, and patient outcomes in MASLD in this narrative review.

## 1. Introduction

The global prevalence of metabolic dysfunction-associated steatotic liver disease (MASLD), previously termed non-alcoholic steatotic fatty liver disease (NAFLD), has risen in parallel with rates of type 2 diabetes and obesity. MASLD is now the most common cause of chronic liver disease and the leading indication for liver transplantation in the United States. Patients with MASLD have an increased risk of progression to metabolic dysfunction-associated steatohepatitis (MASH), cirrhosis, and hepatocellular carcinoma, as well as extrahepatic malignancies and liver- and cardiovascular-related mortality. Diet and lifestyle modifications with a goal of ≥10% total body weight loss—required to reverse steatosis, steatohepatitis, and fibrosis—are often challenging and ineffective. Although novel pharmacotherapies have recently been approved and others are in development, cost, adherence, and adverse effects remain potential limitations [1].

Bariatric surgery, including Roux-en-Y gastric bypass and sleeve gastrectomy, is highly efficacious and a cost-effective treatment for obesity and associated medical problems. In the BRAVES trial, 288 patients with biopsy-proven MASH were randomly assigned to lifestyle modification plus best medical care (n = 96), Roux-en-Y gastric bypass (RYGB) (n = 96), or sleeve gastrectomy (SG) (n = 96). MASH resolution was significantly higher in the RYGB group (56%) and the SG group (57%) compared to the lifestyle modifications group (16%) (*p* < 0.001) [2]. Endoscopic bariatric and metabolic therapy (EBMT) has recently emerged as a promising treatment modality and offers an alternative to surgery. This adds options to the continuum of care for patients with variable risk factors, especially in populations where surgery may be of greater risk and an endoscopic option offers more robust treatment when surgery is not an option. Endobariatrics may be an effective modality in patients with a hostile abdomen, severe associated medical problems that increase operative risk, or as a bridge to surgery in patients with a BMI > 70. EBMT also offers options to patients who are not candidates for bariatric surgery or who decline surgery, but would still benefit from an intervention to augment their dietary and lifestyle efforts.

**Figure 1:**
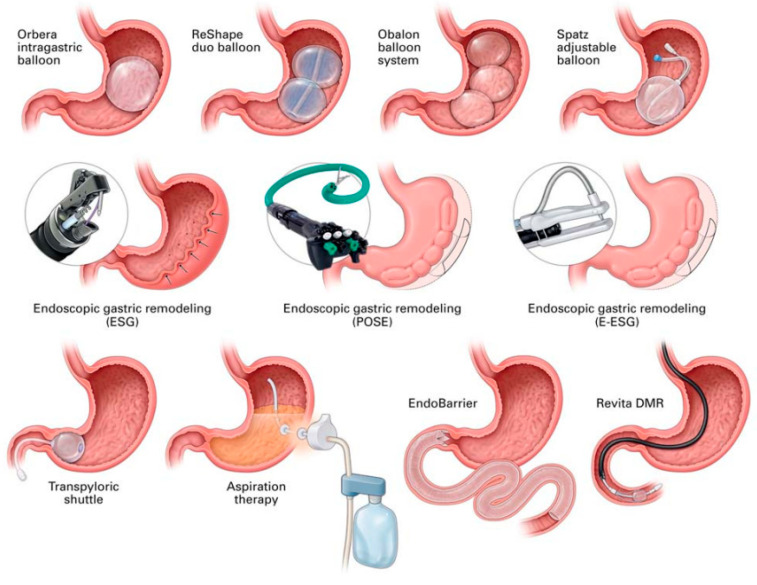
A schematic representation of endobariatric and metabolic therapies. Of note, some of these therapies are not discussed in this manuscript. Abbreviations: ESG—endoscopic sleeve gastroplasty, DMR—duodenal mucosal resurfacing, POSE—primary obesity surgery endoluminal. Image source: [4]. Permission to reuse the image in a journal obtained from RightsLink^TM^ Copyright Clearance Center.

Primary EBMTs have included intragastric balloon, aspiration therapy, endoscopic sleeve gastroplasty, duodenal mucosal resurfacing, duodenal–jejunal bypass liner, and primary obesity surgery endoluminal (POSE 2.0), while secondary EBMTs comprise transoral outlet reduction, argon plasma coagulation, and revisional endoscopic sleeve gastroplasty (Figure 1).

In a systematic review and meta-analysis of 18 studies, EBMTs were associated with an average weight loss of 14.5% of initial weight at 6 months and a significant decrease in liver fibrosis by a standardized mean difference (SMD) of 0.7 (95% CI, 0.1, 1.3; *p* = 0.02). There were significant improvements in ALT (−9.0 U/L; 95% CI, −11.6, −6.4; *p* < 0.0001), hepatic steatosis (SMD: −1.0; 95% CI, −1.2, −0.8; *p* < 0.0001), and histologic NAFLD activity score (−2.50; 95% CI, −3.5, −1.5; *p* < 0.0001). EBMTs were also associated with significantly improved metabolic parameters, such as insulin resistance and waist circumference [3].

We outline the recent literature on primary EBMTs and secondary EBMTs, the associated pathophysiologic mechanisms, safety, and outcomes in MASLD in this narrative review.

## 2. Methods

A narrative review of the literature regarding the endobariatric management of MASLD was conducted. PubMed computerized search and Google Scholar were utilized to find articles with the following title or keywords: “endobariatric”, “metabolic”, “metabolic dysfunction-associated steatotic liver disease”, “intragastric balloon”, “aspiration therapy”, “endoscopic sleeve gastroplasty”, “duodenal mucosal resurfacing”, “duodenal-jejunal bypass liner”, “primary obesity surgery endoluminal (POSE 2.0)”, “transoral outlet reduction”, “argon plasma coagulation”, “revisional endoscopic sleeve gastroplasty”, “MASLD” and “MASH”. Articles were excluded if they were not available in English or full text. Articles that were unrelated to the topic and duplicate articles were removed. Systematic reviews, meta-analyses, and randomized controlled trials were assigned a high priority. A total of 61 articles were included in the final review. The present study does not contain novel studies with human participants or animals conducted by any of the authors. The authors provided original, anonymized radiographic and endoscopic images that serve to highlight the points outlined in the manuscript. Additionally, images from published manuscripts were used, and the source of the images was cited. Permission to reuse the image was obtained accordingly. The authors reviewed all articles within their discipline. The manuscript and images were independently reviewed and revised by all authors.

### 2.1. Primary EBMTs

#### 2.1.1. Intragastric Balloon (IGB)

IGB involves the endoscopic placement of a saline- or gas-filled balloon in the gastric lumen. The IGB decreases the volume of the stomach for food, is associated with delayed gastric emptying, and triggers stretch receptors, which result in satiety and decreased caloric intake. IGB is also associated with hormonal changes. Although the IGB made of polyurethane was first introduced in 1985, termed as the Garren-Edwards Gastric Bubble, it was withdrawn from the market in 1988 due to poor clinical response, spontaneous balloon deflation, and complications, i.e., gastric ulcers, perforations, and intestinal obstructions, some requiring surgery. There have been notable advances in IGB, which include the use of more durable, silicone-based materials. The IGB typically requires approximately 400–600 mL of volume for weight loss, remains in place for 6 months, and is removed endoscopically. There are three FDA-approved IGBs in the US—the Orbera™, Spatz3™, and Obalon™. The Orbera™ (Apollo Endosurgery, Austin, TX, USA) is the only IGB that is commercially available. All three FDA-approved IGBs are indicated for patients with a BMI of 30–40 kg/m^2^ who have failed to respond to lifestyle and nutritional modifications. Common adverse events include nausea, vomiting, and abdominal pain, and typically occur within 2 weeks after IGB placement (Panel 1). Serious, but rare, complications of luminal obstruction, perforation, balloon migration, and balloon hyperinflation from infection have been documented [5].

There have been several studies that explored the use of IGB in patients with MASLD. In a prospective study of 21 patients with early hepatic fibrosis who underwent IGB treatment for 6 months with paired liver biopsies, the mean TBWL was 11.7% ± 7.7%. There was improvement in NAS in 90% of patients with a median decrease of 3 points, as well as fibrosis stage improvement of 1.5 stages in 50% of patients. Half of the patients in the study met the FDA endpoints for MASH resolution and fibrosis improvement. Additionally, there were significant reductions in HbA1c (1.3% ± 0.5%) (*p* = 0.02) and waist circumference (14.4 ± 2.2 cm) (*p* = 0.001) [6].

Similarly, Lee et al. reported 18 patients who underwent IGB placement and had a significant reduction in the mean BMI (1.52 vs. 0.8, *p* = 0.0008) and median NAS (2 vs. 4, *p* = 0.03) as well as an improvement in median steatosis scores at 6 months compared to a sham group. There was no change in median lobular inflammation, hepatocellular ballooning, or fibrosis scores in either group, although the study had a small sample size [7]. Moreover, several additional studies revealed a mean decrease in liver enzymes with IGB [8,9,10,11,12,13]. There were three studies that reported a reduction in hepatic steatosis with IGB [6,14,15].

In a meta-analysis of nine studies, IGB was associated with a decrease in ALT by −10.02 U/L (95% CI, −13.2, −6.8), a decrease in GGT by −9.82 U/L (95% CI, −12.9, −6.8), and a decrease in BMI by −4.98 kg/m^2^ (−5.6, −4.4). There was an improvement in hepatic steatosis from baseline to after 6 months of IGB, which was determined by MRI (fat fraction, 16.7 ± 10.9–7.6 ± 9.8, *p* = 0.003) and ultrasound (severe liver steatosis, 52–4%, *p* < 0.0001). The histological NAS was lower after 6 months of IGB versus control with sham endoscopy and diet (2 ± 0.75 vs. 4 ± 2.25, *p* = 0.03) [16].

Similarly, a meta-analysis of 19 studies and 911 patients revealed that IGB was associated with a decrease in the NAS with a mean difference (MD) of −3.0 [95% CI: −2.41 to −3.59], decrease in ALT, MD of −10.40 U/L [95% CI: −7.31 to −13.49], decrease in the liver volume, MD −397.9 [95% CI: −212.78 to 1008.58] and a decrease in liver steatosis, MD −37.76 dB/m [95% CI: −21.59 to −53.92]. Additionally, there were significant reductions in associated medical problems, including body weight, BMI, glycated hemoglobin, and HOMA-IR [17].

IGBs are a safe and effective therapy for patients with MASLD while the IGB remains in place, resulting in significant improvement in liver enzymes and insulin resistance. Limitations of IGB include limited data on long-term outcomes following balloon removal, preferred duration of balloon placement, complication rates, the durability of the metabolic effects in patients who regain weight, and sustained histologic and biochemical improvement.

#### 2.1.2. Aspiration Therapy

AspireAssist™ (Aspire Bariatrics, King of Prussia, PA, USA) is an endoscopic device for aspiration therapy, which was approved by the FDA in 2016 for a BMI of 35–55 kg/m^2^. It consists of a 30-French aspiration tube connected to an external aspiration device and allows for the removal of approximately 30% of ingested calories from the stomach post-meal. The tube is inserted endoscopically using a standard pull technique. A systematic review of 37 trials involving 15,639 patients revealed that aspiration therapy was associated with a significantly higher TBWL (12.1–18.3%) compared to lifestyle modifications alone (3.5–5.9%) at 12 months. Aspiration therapy was also noted to reduce ALT (−5.2 to −9.8 U/L) and AST (−2.7 U/L) levels (*p* < 0.0001) [18]. AspireAssist™ was discontinued in 2022 when Aspire Bariatrics (King of Prussia, PA, USA) ceased operations due to financial difficulties (Panel 2). Additional studies are warranted to explore the long-term effects of aspiration therapy on MASLD and MASH.

#### 2.1.3. Endoscopic Sleeve Gastroplasty (ESG)

ESG was first performed in 2012 and involves full-thickness plications or sutures to reduce the volume of the gastric lumen and prolong gastric emptying. ESG was approved by the FDA in 2022 for patients with a BMI of 30–50 kg/m^2^ [19]. The MERIT trial, which compared lifestyle modifications versus ESG with a follow-up period of two years, demonstrated a TBWL of 14.7% and significant improvements in metabolic outcomes with ESG [20]. In a subset of patients from the MERIT trial, Vargas et al. conducted a comparative study with a total of 36 patients. Sixteen patients were randomized to ESG, and seventeen patients were randomized to lifestyle interventions alone. The time for 50% of the contents of the stomach to empty was significantly delayed in the ESG group compared with the lifestyle interventions group at 3 months and remained delayed in the ESG group at 12 months. Fasting ghrelin, glucagon-like peptide 1, and polypeptide YY levels were significantly increased 18 months after ESG [21]. ESG has also been associated with an increase in leptin [22].

In a meta-analysis of eight studies with a total of 1772 patients who underwent ESG, the mean TBWL of 15.1% at 6 months. Weight loss was sustained at 12 months and 18–24 months with a TBWL of 16.5% and 17.2%, respectively. Severe adverse events were reported in 2.2% of patients and included pain or nausea requiring hospitalization (1.08%), upper gastrointestinal bleeding (0.56%), and peri-gastric leak or fluid collection (0.48%) [23].

With regard to outcomes in MASLD, Espinet Coll et al. reported in a study with 30 patients with MASLD, of which 15 patients underwent IGB for 1 year, and 15 patients underwent ESG. ESG was associated with an average TBWL of 16.34% at 12 months, a decrease in the fatty liver index, hepatic steatosis index, NAFLD fibrosis score, hepatic ultrasonographic steatosis, subcutaneous fat (*p* < 0.001), as well as the HOMA-IR, insulin, and triglycerides (*p* < 0.05) after 12 months [24].

Sharaiha et al. [25] conducted a study with 118 patients with MASLD and a mean BMI of 40 ± 7 kg/m^2^ who underwent ESG. The mean TBWL was 15.5% (95% confidence interval, 13.3–17.8%) at 2 years. There was an improvement in the hepatic steatosis index score, which decreased 4 points per year (*p* ≤ 0.001), and an improvement in the NAFLD fibrosis score of 0.3 points per year (*p* = 0.034). Approximately 20% of patients improved from F3–F4 or indeterminate to F0–F2. The HOMA IR significantly improved after only 1 week from ESG (*p* = 0.019) and continued to improve up to 2 years (*p* = 0.03) [26]. Similarly, Sharaiha et al. conducted a study of 91 patients who underwent ESG. The patients’ mean BMI before ESG was 40.7 ± 7.0 kg/m^2^. ESG was associated with TBWL of 14.4% at 6 months, 17.6% at 12 months, and 20.9% at 24 months. Patients had a statistically significant decrease in hemoglobin A1c (*p* = 0.01), systolic blood pressure (*p* = 0.02), waist circumference (*p* < 0.001), alanine aminotransferase (*p* < 0.001), and serum triglycerides (*p* = 0.02) at 12 months after ESG. There was no significant change in low-density lipoprotein before or after ESG. One patient was noted to have had a perigastric leak, which was managed non-operatively [25].

A study of 99 patients who underwent ESG found that the TBWL was 16.6 ± 7.4% at six months and 16.6 ± 9.6% at one year. There was a reduction in AST and ALT, which was statistically significant at six and twelve months after ESG. The study revealed a significant reduction in the rates of T2DM, hypertension, GERD, OSA, and dyslipidemia (*p* < 0.001). Additionally, ESG was associated with a significant reduction in blood glucose between the pre-operative period and the 6-month and 12-month follow-up [27].

In a meta-analysis of 4 studies and 175 patients, ESG was associated with a significant (*p* < 0.05) reduction in hepatic steatosis index of 4.85 (95% CI −6.02, −3.67), 0.5 in NAFLD fibrosis score (95% CI −0.80, −0.19), 6.32 U/L in ALT (95% CI −9.52, −3.11), 17.28% in TWL (95% CI −18.24, −16.31), 6.31 kg/m^2^ in BMI (95% CI −8.11, −4.52), 47.97% in EWL (95% CI −49.10, −46.84), and 0.51% in HbA1c (95% CI −0.90, −0.12) [28].

A recent systematic review and meta-analysis of 10 studies and 4320 patients evaluated the impact of ESG on T2DM and metabolic syndrome. ESG was associated with significant improvements in T2DM, hyperlipidemia, and hypertension, as well as significant decreases in hemoglobin A1c, fasting glucose, HOMA-IR, LDL cholesterol, and triglycerides at 12 months. Disease improvement was defined by discontinuation of some or all related medications [29]. While these findings support the metabolic efficacy of ESG during short-to-intermediate follow-up, long-term durability beyond one year and effects on hepatic histology are not known.

Long-term outcomes following ESG were evaluated in a prospective cohort of 404 patients who underwent ESG as monotherapy, with a follow-up of 5 years. The study demonstrated significant and sustained improvements in metabolic and hepatic biomarkers, including reductions in hemoglobin A1c, systolic blood pressure, and ALT levels at 5 years. Total body weight loss was maintained over time, with 11.8% TBWL at 60 months, among patients with available follow-up. Importantly, improvements in liver enzymes suggest a favorable effect on MASLD. However, the evaluation was limited to biochemical markers with no histologic endpoints. While this study provides important evidence of the durability of metabolic effects after ESG, attrition rate and the lack of imaging or biopsy-based liver outcomes limit conclusions with regard to long-term disease modification [30].

A prospective, propensity-score-matched case–control study compared ESG with semaglutide in patients with MASLD over one year. ESG was associated with significantly greater total body weight loss compared with semaglutide. There were improvements in ALT and AST in both groups, with no significant between-group differences. However, among patients with elevated baseline fibrosis risk (FIB-4 > 1.3), semaglutide resulted in greater improvement in FIB-4 scores compared with ESG, and only semaglutide demonstrated significant within-group improvement in FIB-4 over time [31]. These findings suggest that while both ESG and GLP-1 receptor agonist therapy improve hepatic biochemical markers in MASLD, pharmacotherapy may confer additional benefit in fibrosis risk reduction, whereas ESG provides superior weight loss at one year.

In a retrospective case series, ESG after LT was associated with a median TBWL of 27.2% (IQR, 8.0–30.0%) at 18.8 months follow-up with 5 postoperative adverse events in 4 patients, which were mild. The liver graft function was stable. Additional studies are warranted to explore ESG after LT [32].

Limitations of ESG include cases of portal hypertension, esophagogastric varices, coagulopathy, large hiatal hernia (>3–5 cm), active peptic ulcer disease, neoplastic lesions, or a prior gastric surgery [33].

ESG is associated with clinically significant improvements in weight loss, metabolic parameters, and liver biochemistries in patients with MASLD (Table 1). Emerging long-term data suggest the durability of metabolic and liver enzyme improvements following ESG, with sustained weight loss up to five years. However, evidence for improvement in fibrosis risk and histologic endpoints remains limited, and comparative data suggest that pharmacologic therapies may offer greater benefit for fibrosis-related markers in selected patients. Further prospective studies, which incorporate imaging and histologic outcomes, are needed to define the role of ESG as a disease-modifying therapy in MASLD and provide information regarding the integration of ESG with medical therapy. Overall, ESG is a safe and efficacious procedure for patients with MASLD. ESG in combination with one of the FDA-approved medications, i.e., resmetirom or semaglutide, for MASH, may offer a promising alternative. Additional studies are warranted on the topic.

#### 2.1.4. Primary Obesity Surgery Endoluminal (POSE)

While ESG has been demonstrated to be safe and effective, suture loss is a limitation. POSE allowed for improved plication durability and reproducibility. POSE was first described in 2011 in Barcelona, Spain, and targeted the gastric fundus and involved a few plications in the distal body of the stomach (Table 2) [34]. However, in the ESSENTIAL trial, POSE failed to meet the weight loss endpoints [35]. More recently, in 2019, POSE 2.0 was introduced to overcome the limitations of the previous procedure. POSE 2.0 involves an incisionless operating platform (USGI Medical, San Clemente, CA, USA) to shorten and narrow the stomach using multiple, full-thickness plications in the gastric body and durable suture anchor pairs, which results in a tubular stomach, decreased gastric volume, decreased gastric emptying, and weight loss [36]. Adverse events have been reported to be infrequent, approximately 2.5–5%, and include nausea and pain. Gastrointestinal perforation or gastrointestinal bleeding is rare. In a study of 44 patients by Nava et al. with a mean BMI of 37, POSE 2.0 was associated with a mean TBWL of 12.2%, a mean change in ALT of −14, and a mean change in AST of −4.8 at 6 months. There was significant improvement in the steatosis stage at 12 and 24 months and significant improvement in the fibrosis stage at 24 months [36]. In a trial of 20 patients who underwent lifestyle modifications with POSE 2.0 and 22 patients who underwent lifestyle alone, POSE 2.0 was associated with a significant improvement in Controlled Attenuated Parameter (CAP) from 322.6 to 235.5 dB/m (*p* < 0.001) [37]. There was an improvement in liver enzymes and AST-to-platelet ratio at 12 months. There was a significantly higher resolution of steatosis and %TBWL in the POSE 2.0 group compared with the control group. In a meta-analysis of 4 studies, POSE 2.0 was associated with improvements in HbA1c and cholesterol. While there were improvements in AST and ALT, the difference was not statistically significant, and there was high heterogeneity [38]. POSE 2.0 is a safe and efficacious procedure. Limitations of POSE 2.0 include cost and gastric remodeling, which may complicate future revisional bariatric surgery [39]. Additional studies are warranted to explore POSE 2.0 in patients with large cohorts of patients with MASLD.

#### 2.1.5. Duodenal Mucosal Resurfacing (DMR)

DMR involves the circumferential hydrothermal ablation of the duodenal mucosa distal to the ampulla of Vater to the ligament of Treitz. DMR affects abnormal nutrient sensing and neurohormonal mechanisms associated with MASLD, as well as regenerates the epithelium. DMR was first introduced in 2016 for patients with type 2 diabetes mellitus. Rajagopalan et al. demonstrated that a single session of DMR resulted in clinically significant improvement in hyperglycemia in patients with type 2 diabetes mellitus with acceptable safety and tolerability [40]. In the REVITA-2 trial, the safety and efficacy of DMR on glycemic control and liver fat content were evaluated in patients with T2DM. The study demonstrated that there was a greater reduction in HbA1c in the DMR group versus the sham group and greater 12-week liver fat change in the DMR group compared to the sham group. Most adverse events were mild and transient [41]. Van Baar et al. documented in a multicenter study of 36 patients, improvements in HbA1c (−10 ± 2 mmol/mol (−0.9% ± 0.2%), *p* < 0.001), fasting plasma glucose (−1.7 ± 0.5 mmol/L, *p* < 0.001) and HOMA-IR at 24 weeks post-DMR (−2.9 ± 1.1, *p* < 0.001). There was a moderate reduction in weight (−2.5 ± 0.6 kg, *p* < 0.001), and liver aminotransferase levels decreased. These effects were sustained for 12 months [42]. Hadefi et al. reported that DMR was not associated with MASH resolution, and there was improvement in fibrosis with no worsening of MASH in 27% of patients. However, the study had a small sample size, and the population was heterogeneous [43]. There are several possible limitations of DMR. There is limited data on long-term metabolic improvements. Generally, patients with gastric bypass are excluded. DMR may not be suitable for patients with Crohn’s disease, celiac disease, or chronic pancreatitis. Certain duodenal abnormalities may preclude DMR, including ulcers, polyps, varices, or strictures. Incomplete resurfacing may occur in cases of duodenal tortuosity.

#### 2.1.6. Duodenal–Jejunal Bypass Liner (DJBL)

DJBL, also known as EndoBarrier™, is a 60 cm, impermeable endoscopic implant first reported in 2008, which is anchored in the duodenal bulb and extends to the proximal jejunum [45]. DJBL results in the bypass of nutrients in the proximal small intestine and mimics the duodenal–jejunal exclusion portion of Roux-en-Y physiology without permanent surgical alterations [46]. DJBL is reversible, typically does not require general anesthesia, and patients may be discharged the same day. However, fluoroscopy is often needed, and it is recommended that patients take proton pump inhibitors and a liquid diet prior to placement and shortly thereafter.

In a study of 32 patients who underwent DJBL for 48 weeks, there was a statistically significant change in the AST and ALT with a factor of 0.74 and 0.63, respectively. There was a statistically significant difference of −0.21 (−0.28 to −0.13) for FAST. A slight improvement in FIB-4 was noted, but there were no changes in fibrosis based on liver stiffness measurements, NFS, and ELF. Eight DJBL were extracted early due to device-related complications, and eight complications led to hospitalization [47].

Gollisch et al. found that EndoBarrier therapy reduced liver stiffness from 10.4 kPa at baseline to 5.3 kPa after 1 year (*p* < 0.01), and there was a slight improvement in CAP from 343 dB/m to 317 dB/m (*p* < 0.05), although most patients still had advanced steatosis [48].

In a meta-analysis of ten RCTs, DJBL was associated with superior excess weight loss and greater decrease in HbA1c compared to the control group [49].

Limitations of DJBL include serious adverse events, including hepatic abscesses, gastrointestinal hemorrhage, and device migration. A randomized controlled trial in 2015 was discontinued due to a hepatic abscess rate of 3.3% (https://www.clinicaltrials.gov/study/NCT01728116, accessed on 15 December 2025). Additionally, metabolic improvements may wane post-removal. A new trial exploring outcomes with DJBL, the STEP-1 trial, was approved by the FDA in 2019 (https://clinicaltrials.gov/study/NCT01728116, accessed on 15 December 2025), but the results have not been reported.

### 2.2. Secondary EBMTs

#### 2.2.1. Transoral Outlet Reduction

Transoral outlet reduction (TORe) is a secondary EBMT that reduces the diameter of a dilated gastrojejunal anastomosis following RYGB, a common etiology for weight regain or inadequate restriction. In a meta-analysis of 12 studies and 1154 patients, TORe was associated with a mean total weight loss of 8.9% (95% confidence interval [CI], 6.5–11.3%; *p* < 0.0001). Weight-loss outcomes at 12 months were comparable between suturing-based and plication-based TORe techniques. Subgroup analyses demonstrated greater weight loss with a purse-string suture pattern compared with non-purse-string configurations. The overall adverse event rate was low at 1.5% (95% CI, 0.8–2.6%; *p* < 0.0001) [50]. In a retrospective cohort of 50 patients who underwent TORe for weight regain, there were significant improvements in ALT, AST, FIB-4 score, and liver stiffness on transient elastography. Mean total weight loss was 8.8 ± 11.2%, and there were significant improvements in hemoglobin A1c and insulin resistance by HOMA-IR [51]. TORe may confer metabolic and hepatic benefits in patients with MASLD following Roux-en-Y gastric bypass, likely through modest, but clinically significant weight loss and improvements in insulin resistance. Limitations of TORe include cases of weight regain unrelated to dilated gastrojejunal anastomosis and patients with anastomotic ulcers/inflammation or a large hiatal hernia. There may be a need for several TORe procedures, and associated conditions, i.e., dumping syndrome, may be only partially resolved. Additionally, there is limited long-term data on metabolic improvements.

#### 2.2.2. Argon Plasma Coagulation

Argon plasma coagulation (APC), a noncontact electrocoagulation method, has been demonstrated to be efficacious in decreasing the size of a dilated gastrojejunal anastomosis following RYGB, similar to TORe [53]. Limitations include the possible need for multiple sessions of APC and limited data on APC and liver-related outcomes.

#### 2.2.3. Revisional Endoscopic Sleeve Gastroplasty

Weight recidivism has been reported to be approximately 14–37% after laparoscopic sleeve gastrectomy and has been associated with sleeve dilation [55,56].

Revisional endoscopic sleeve gastroplasty (RESG) is a secondary EBMT that may be utilized for weight regain after bariatric surgery. In a study of 22 patients who underwent RESG for weight regain, most cases followed SG. The mean BMI loss and %EWL were 4.2 kg/m^2^ (±4.7) and 53.1% (±17), respectively [57]. A study by Bahdil et al. reported higher TBWL% with RESG compared to GLP-1/GIP-RA at 3 (11.2% vs. 4.3%, *p* < 0.001), 6 (13.5% vs. 6.8%, *p* < 0.001), and 12 months (13.4% vs. 9.2%, *p* = 0.07) [52]. Studies that report RESG and outcomes in MASLD are warranted.

### 2.3. Future Directions

The combination of EBMTs with pharmacologic therapies—including glucagon-like peptide-1 receptor agonists and thyroid hormone receptor-β agonists approved for MASH with F2-3 fibrosis—may confer additive or complementary benefits. However, the optimal role of EBMTs, whether as standalone therapy, in combination with pharmacologic agents, or as a bridge to bariatric surgery, is not known.

Prospective trials directly comparing EBMTs, pharmacologic therapies, and combination strategies are needed to define optimal sequencing, patient selection, and the durability of response. Studies that incorporate imaging and histologic endpoints will further elucidate mechanisms of action, long-term safety, and the extent to which EBMTs can modify the natural history of MASLD.

## 3. Conclusions

EBMTs have demonstrated not only durable weight loss but also improvements in metabolic parameters, with emerging data supporting their efficacy in hepatic and cardiometabolic outcomes in patients with MASLD. Among the primary EBMTs, there are several studies that document IGB placement and improved outcomes in MASLD, although there is a concern about whether long-term effects following IGB removal are sustained. ESG has a moderate amount of data regarding long-term improvement in metabolic parameters and MASLD. While DMR and POSE 2.0 are promising modalities, additional studies are warranted. Aspiration therapy was discontinued in 2022 when Aspire Bariatrics (King of Prussia, PA, USA) ceased operations due to financial difficulties. DJBL was discontinued in the United States due to adverse events in a clinical trial.

Among the secondary EBMTs, there are several studies that document TORe and weight loss, and a single retrospective study that demonstrated improvement in MASLD. Additional studies are warranted for APC and RESG.

It is important to note that endobariatrics should be considered in the setting of multidisciplinary management of obesity and MASLD, and involve collaboration with hepatology, bariatric endoscopy, bariatric surgery, and nutrition teams. Finally, insurance approval for endobariatric procedures may be a barrier for some patients.

## Figures and Tables

**Table 1:** Primary and secondary endobariatric and metabolic therapies, their duration, estimated total body weight loss, and effect on metabolic dysfunction-associated steatotic liver disease outcomes.

Endobariatric Procedure	Duration	Estimated TBWL	Effect on MASLD	Nature of Evidence
**Primary endobariatric metabolic therapies**				
Intragastric balloon	6 months	8.8–14.1%	Reduced aminotransferases Decreased hepatic steatosis	Retrospective/prospective study, clinical trial, meta-analysis [6,7,8,9,10,11,12,13,14,15,16,17]
Aspiration therapy	Temporary	12.1–18.3%	Reduced aminotransferases	Meta-analysis [18]
Endoscopic sleeve gastroplasty	Permanent	15.1% at 6 months 17.2% at 18–24 months	Reduced aminotransferases Decreased hepatic steatosis and fibrosis	Retrospective/prospective study, clinical trial, meta-analysis [19,20,21,22,23,24,25,26,27,28,29,30,31,32]
Primary obesity surgery endoluminal 2.0	Permanent	14.8–15.7% at 12 months	Reduced aminotransferases Decreased hepatic steatosis and fibrosis	Prospective study, clinical trial, meta-analysis [34,35,36,37,38,39]
Duodenal mucosal resurfacing	Permanent	5%	Reduced aminotransferases Decreased hepatic steatosis and fibrosis	Prospective study, clinical trial [40,41,42,43]
Duodenal–jejunal bypass liner	12 months	8.9–12.6% [44]	Reduced aminotransferases Reduced hepatic steatosis and fibrosis	Retrospective/prospective study, clinical trial, meta-analysis [45,46,47,48,49]
**Secondary endobariatric metabolic therapies**				
Transoral outlet reduction	Permanent	8.8% [50]	Reduced aminotransferases Reduced hepatic stiffness	Retrospective study, meta-analysis [50,51]
Revisional endoscopic sleeve gastroplasty	Permanent	13.4% [52]	Not reported	Clinical trial [53]
Argon plasma coagulation	Permanent	6–10% [54]	Not reported	Retrospective/prospective study, meta-analysis [52,55,56,57]

**Table 2:** Primary and secondary endobariatric procedures, presumptive mechanism of action, changes in hormones, and adverse events.

Endobariatric Procedure	Mechanism of Action	Hormonal Changes	Adverse Events
**Primary endobariatric metabolic therapies**			
Intragastric balloon	Decreased gastric volume Delayed gastric emptying	Decrease in plasma leptin Transient elevation of plasma ghrelin [58]	Common—nausea, vomiting, abdominal pain Rare—luminal obstruction, perforation, balloon migration, balloon hyperinflation from infection of gas-producing organisms of intragastric balloon fluid
Aspiration therapy	Remove 25–30% of calories after a meal	Not established	Abdominal pain Peristomal granulation tissue Peristomal irritation Rare—intra-abdominal fluid collection or infection
Endoscopic sleeve gastroplasty	Decreased gastric volume Delayed gastric emptying	No change in glucagon-like peptide 1 or polypeptide YY No compensatory rise in ghrelin Decreased leptin	Pain, nausea, upper gastrointestinal bleeding, peri-gastric leak or fluid collection
Primary obesity surgery endoluminal 2.0	Decreased gastric volume Decreased gastric emptying	Improvement in glucose/insulin ratio Decrease in postprandial ghrelin Increase in postprandial peptide YY [59]	Nausea and pain Rare—gastrointestinal perforation, gastrointestinal bleeding
Duodenal mucosal resurfacing	Hydrothermal ablation of duodenal mucosa affects abnormal nutrient sensing, regenerates epithelium	Reset and correct abnormal signaling arising from the duodenal mucosa, improved function of pancreatic β cells, and improved glucose tolerance	Abdominal pain and diarrhea
Duodenal–jejunal bypass liner	Reversible, 60 cm impermeable endoscopic implant results in bypass of nutrients in proximal small intestine, mimics Roux-en-Y physiology	Increased GLP-1 and PYY secretion [60]	Abdominal pain, nausea, vomiting Hepatic abscesses
**Secondary endobariatric metabolic therapies**			
Transoral outlet reduction	Reduction in the size of gastrojejunal anastomosis	Delay in the decrease in ghrelin levels Decrease in GLP-1 levels [61]	Abdominal pain, bloating, nausea Rare—bleeding, perforation, infection, stenosis
Revisional endoscopic sleeve gastroplasty	Reduction in the size of the stomach	Not established	Pain, nausea, upper gastrointestinal bleeding, peri-gastric leak or fluid collection
Argon plasma coagulation	Reduction in the size of the gastrojejunal anastomosis, delay in gastric emptying, increased satiety	Possible increase in peptide YY [61]	Vomiting, upper GI bleeding, ulcer, leak, stenosis

## Data Availability

Not applicable for this study.

## List of Abbreviations

AST—aspartate aminotransferases, ALT—alanine aminotransferase, BMI—body mass index, CAP—Controlled Attenuation Parameter, CI—confidence interval, DJBL—duodenal–jejunal bypass liner, FDA—Food and Drug Administration, HSI—hepatic steatosis index, HbA1c—hemoglobin A1c, IGB—intragastric balloon, EBMT—endoscopic bariatric and metabolic therapy, EBWL—estimated body weight loss, ELF—Enhanced Liver Fibrosis score, ESG—endoscopic sleeve gastroplasty, FAST—FibroScan-AST score, FIB-4—Fibrosis Index, kPa—kilo pascal, GERD—gastroesophageal reflux disease, HOMA-IR—Homeostatic Model Assessment of Insulin Resistance, MASLD—metabolic dysfunction-associated steatotic liver disease, MASH—metabolic dysfunction-associated steatohepatitis, MD—mean difference, NAFLD—non-alcoholic steatotic fatty liver disease, NAS—Non-Alcoholic Fatty Liver Disease Activity Score, NFS—Non-Alcoholic Fatty Liver Disease Fibrosis Score, OSA—obstructive sleep apnea, POSE—primary obesity surgery endoluminal, RYGB—Roux-en-Y gastric bypass, SG—sleeve gastrectomy, SMD—standardized mean difference, TBWL—total body weight loss.
